# Podcast-based learning: report of podcast as an education tool for residents in the emergency room

**DOI:** 10.1590/acb404525

**Published:** 2025-07-07

**Authors:** Diego Adão, Wagner Gomes da Nóbrega Silva, Parisina Fraga Carvalho, Gabriela Caetano Lopes Martins, Georges Badin Hofmeister, Karin Romano Posegger, Adriano Meyer Pflug, Carlos Augusto Metidieri Menegozzo

**Affiliations:** 1 Universidade Federal de São Paulo – Escola Paulista de Medicina – Departamento de Cirurgia – São Paulo (SP) – Brazil.; 2 Universidade de São Paulo – Hospital das Clínicas – Departamento de Cirurgia – São Paulo (SP) – Brazil.

**Keywords:** Education, Medical, Webcasts as Topic, Webcast, Internship and Residency, General Surgery

## Abstract

**Purpose:**

To describe the academic utilization of podcasts as an educational tool for general surgery and emergency medicine residents.

**Methods:**

This narrative report details a one-year group experience incorporating podcasts into the curriculum of the general surgery and emergency medicine residency programs, focusing on podcast-based learning within the academic emergency department of a quaternary university hospital in an urban center in São Paulo, Brazil. The authors, comprising podcast developers, preceptors, and residents, shared their experiences with implementing and utilizing the podcast in the residency curriculum.

**Results:**

Over the course of one year, 50 episodes of the podcast “Mania de Cirurgia” were delivered to residents rotating in the emergency room. The episode content consisted of 64% clinical topics, 12% summaries of scientific events, 12% interviews with specialists, and 12% soft skills development. The authors reported that both residents and preceptors expressed high satisfaction with the podcast’s integration as a consistent educational tool in the curriculum.

**Conclusion:**

The incorporation of a podcast as an educational tool was enthusiastically received by both residents and preceptors. Podcasts may serve as a valuable complement to traditional teaching methods, enabling updates on general surgery topics, fostering evidence-based practice, and enriching professional experiences.

## Introduction

Medical education has undergone significant transformations over the last decades. In the 20th century, the Flexner report proposed the systematization of medical education based on the recognition of shortcomings, proposing an organized curriculum grounded in the biomedical model^1^.

In recent years, particularly post-COVID-19 era, medical education has been revolutionized with the rise of digital media platforms, asynchronous educational methods, and active learning replacing traditional in-person teaching^2^. These modalities are accepted among newer generations, such as generations Alpha and C^3^. Free open-access medical education (FOAMed) stands out, consisting of freely available audiovisual educational content such as podcasts, blogs, and video lectures^4^.

North America registered approximately 143.3 million podcast listeners in 2023 (129.9 million in the United States of America)^5^. Latin America had 135.2 million listeners, with Brazil leading, at 51.8 million. Projections indicate that Latin America will surpass North America in 2025, reaching 157.8 million listeners, reflecting a global trend in podcast consumption, including in healthcare.

This study aimed to describe a personal and academic experience with implementing a surgery podcast as an educational tool through podcast-based learning.

## Methods

This narrative report details the authors’ experience utilizing a surgery podcast as an andragogical tool within the surgical emergency setting, incorporated into the curriculum of the general surgery and emergency medicine residency programs. The study was conducted in the surgical emergency department of a quaternary university hospital in an urban center in Brazil (Escola Paulista de Medicina, Universidade Federal de São Paulo) from January 2024 to December 2024.

The podcast “Mania de Cirurgia” is an academic initiative led by three acute care surgeons (AMF, CAMM, and DA). This nonprofit and freely accessible podcast focuses on surgical topics while upholding ethical and confidentiality standards. Episodes are recorded remotely via the Zencastr platform (Inc., 2014) and edited using Audacity software (Muse Group, The Audacity Team, 2000). Launched in September 2022, the podcast has released over 130 episodes, each averaging 50 to 60 minutes and including references at the end. It is available on streaming platforms such as Spotify (Stockholm, 2006), Deezer (Paris, 2007), and others (see https://open.spotify.com/show/7xlDN1tt4OcMhwqXqBjEVL). Episodes are organized into four categories: clinical topics, summaries of scientific events, interviews with specialists, and soft skills development.

The podcast listeners included preceptors and residents from all three years of the general surgery and emergency medicine residency programs during their emergency department rotations. Each rotation averaged 40 days, with podcast episodes tailored to either the real-time demands of cases encountered in daily practice or pre-selected topics. Episodes were provided under preceptor guidance, with links shared in a group chat following academic rounds to facilitate discussion at subsequent meetings.

The impressions documented in this study reflect the perspectives of the preceptors and residents, who are also authors, based on their experiences with the podcast and informal discussions with colleagues.

## Results

Over the course of one year of academic rounds, more than 70 residents from the general surgery and emergency medicine programs engaged with at least 50 episodes of the podcast “Mania de Cirurgia.” Most episodes, selected by the preceptorship, focused on clinical content, reflecting the prevalence of surgical conditions encountered in the emergency setting. The distribution of episodes provided to residents by content type was as follows: 64% clinical topics, 12% summaries of scientific events, 12% interviews with specialists, and 12% soft skills development. For a comprehensive overview of the topics covered in the first 100 episodes (Table 1).

**Table 1:** Themes of “Mania de Cirurgia” podcast first 100 episodes segmented by content type.

Type of content	Subject	Number of episodes
Clinical	Abdominal wall closure	1
	Acute appendicitis	1
	Acute cholecystitis	3
	Acute diverticulitis	1
	Acute pancreatitis	2
	Adynamic ileus	1
	Bariatric surgery	2
	Colorectal cancer	1
	Crohn's disease	1
	Devices	2
	Esophagus	1
	Fournier's gangrene	1
	Gastrointestinal bleeding	2
	Hernia	3
	ICU in general surgery	1
	Melanoma	1
	Nutrition	1
	Ostomies	2
	Patient blood management	1
	Peptic ulcer	1
	Perioperative care	1
	Peritoneostomy	1
	Surgical prescription	2
	Surgical technique	10
	Trauma	19
	Tubes and drains	2
	Vascular acute abdomen	2
Interviews with specialists		12
Scientific events	São Paulo Congress of Surgery 2022	1
	Cirurgião Ano 13	1
	35th Panamerican Congress of Trauma, Critical Care and Emergency Surgery	1
	ACS congress 2023	1
	Cirurgião Ano 14	1
	ASCO 2024	1
Soft Skills	Career	5
	Choosing a residency program	1
	Ethics and laws	4
	Handoff process	1
	Learning	2
	Residency interview	1
	Science	2
Total episodes		100

Source: Elaborated by the authors.

Based on the authors’ reflections, the podcast “Mania de Cirurgia” proved to be an accessible, practical, and effective educational tool within the academic framework of a university-based surgical residency program, introducing podcast-based learning as a novel and innovative teaching technique. When episodes were aligned with real-time patient cases encountered during emergency rounds, this approach enhanced the relevance and impact of learning, making it more engaging and meaningful for residents. The contextual integration of podcast-based learning not only facilitated deeper understanding but also encouraged active participation and discussion among learners. The flexibility of accessing episodes on-demand via widely available streaming platforms further supported its seamless incorporation into the demanding schedules of residents and preceptors. Overall, the authors viewed this one-year experience as highly positive, highlighting podcasts’ potential to complement traditional teaching methods, enrich resident education, and foster a collaborative learning environment in the high-pressure setting of surgical emergency care.

## Discussion

Our experience represents the first documented case of integrating podcasts as a formal teaching tool within the curriculum of general surgery and emergency medicine programs. The use of podcast-based learning was enthusiastically embraced by both preceptors and residents, who found it to be a valuable curricular resource that significantly enhanced their understanding of clinical cases discussed. To our knowledge, no data exists on the use of medical podcasts in Brazil. In contrast, a 2024 report from the United States of America identified 50 medical podcasts, with 80% focusing on clinical knowledge^6^.

While highly valuable, these podcast episodes are not intended to replace traditional lectures but rather to complement them, enriching the educational experience within the residency curriculum. Episodes focusing on clinical content were the most frequently shared and recommended as supplementary resources. For example, an episode on acute cholecystitis explored indications for subtotal cholecystectomy in complex gallbladder cases, directly relating to a patient treated in the emergency department. This allowed residents to deepen their understanding of the disease’s pathophysiology and engage with key discrepancies in the literature. Scientific references provided in the episode descriptions were discussed with attending physicians during clinical rounds, creating an interactive experience akin to a weekly congress.

Episodes summarizing scientific events played a vital role in keeping residents updated. The demanding nature of residency often limited their ability to attend such events in person. The podcast bridged this gap by delivering insights from major national and international conferences, enabling residents to stay informed and review critical topics.

Episodes featuring interviews with specialists offered residents the opportunity to hear from leading professionals in the field, gaining insights into their daily routines and experiences across public and private healthcare settings. These discussions provided valuable perspectives on career paths and clinical practice.

Episodes addressing soft skills covered often-overlooked topics, such as career decision-making and delivering difficult news. Notably, episodes exploring the I-PASS^7^ system and the Hierarchy of Communication Needs^8^, both standardized approaches to handoff communication, sparked in-depth and engaging discussions among listeners.

Currently, FOAMed is widely disseminated as a curricular teaching strategy in United States of America medical residency programs, especially in emergency medicine, as 97% of all residency programs in this specialty employ them in their schedules^9^. A scoping review demonstrated that podcasts are highly accepted among students and academics, particularly due to their low or no cost to listeners, flexibility, and ability to meet self-defined learning needs^4^. It was observed that 55 to 90% of physicians exposed to podcasts reported changes in their clinical practices. Regarding long-term content retention in residents exposed to podcasts compared to traditional educational interventions, equal or superior performance was observed in podcast-exposed groups^4^.

Ratliff et al.^10^ assessed the impact of the curricular use of podcasts associated with clinical discussions in a neurology residency. A statistically significant increase in residents’ confidence in medical decision-making was observed in two out of three types of medical emergencies evaluated. Up to 94% of residents reported that podcasts were helpful in their learning. O’Neill et al.^11^ evaluated the educational outcomes of general surgery podcasts for undergraduate students. In 47 students, multiple-choice questions were administered pre- and post-exposure to the podcast, revealing an absolute mean increase in baseline scores of 20.3%, which was statistically significant (95% confidence interval 14.67–25.6, *p* < 0.001).

This narrative report has several limitations that warrant consideration. Firstly, we did not formally evaluate the effectiveness of podcast-based learning as an educational method, nor did we employ standardized tools to measure resident satisfaction or knowledge acquisition. The absence of structured interviews or surveys limits our ability to quantify the podcast’s impact on learning outcomes. Additionally, without specific guidelines on optimal listening conditions, distractions during playback could compromise comprehension, as residents often multitask in demanding clinical environments. Most episodes were recorded in Portuguese, which restricts accessibility for non-Portuguese-speaking audiences and limits the podcast’s global reach. Although the episodes were designed for educational purposes and not for diagnostic or therapeutic guidance, there is a risk that some listeners might misinterpret or inappropriately apply the discussed content in clinical practice. Lastly, our report reflects a single-center experience without a control group, making it challenging to generalize findings.

To address these limitations, future research should prioritize controlled and comparative studies to rigorously assess the efficacy of podcasts as a learning tool in medical education. Such studies could incorporate validated assessment tools to measure knowledge retention, clinical application, and learner engagement. Exploring strategies to optimize the listening experience, such as recommending dedicated time for podcast consumption, could enhance comprehension. Producing episodes in additional languages or with subtitles could broaden accessibility. Furthermore, clear disclaimers emphasizing the educational intent of the content may help mitigate the risk of misapplication. Expanding this approach to other institutions and specialties could also provide valuable insights into the scalability and adaptability of podcast-based learning in diverse medical training contexts.

Universities and educational institutions can leverage podcasts featuring faculty members and prominent surgical professionals to enhance scientific education. Given the emphasis on evidence-based medicine and entrustable professional activities-based curricula, fostering critical thinking skills is paramount. Podcasts may serve as a valuable complement to traditional teaching methods, providing accessible updates on general surgery topics and promoting evidence-based practice.

## Conclusion

The incorporation of a podcast as an educational tool was enthusiastically embraced by both residents and preceptors. Podcasts may serve as a valuable complement to traditional teaching methods, enabling timely updates on general surgery topics, fostering evidence-based practice, and enriching professional experiences while promoting engagement in the learning process.

## Data Availability

Data sharing is not applicable.
